# A qualitative study on decent work perception among male nurses in the ICU

**DOI:** 10.3389/fpubh.2026.1942420

**Published:** 2026-10-16

**Authors:** Xianyong Liu, Mengjun Sun, Jiahang Li, MioLeng Au

**Affiliations:** 1 Kiang Wu Nursing College of Macau, Macao, Macao SAR, China; 2 Nursing School of Hunan Normal University, Changsha, Hunan, China

**Keywords:** decent work, intensive care units, job satisfaction, male nurses, qualitative research

## Abstract

**Objective:**

To explore the lived experiences of decent work perception among male ICU nurses in China, so as to provide evidence for nursing administrators to improve management strategies and retain male nursing workforce.

**Methods:**

An interpretative phenomenological research design guided by the Psychology of Working Theory was adopted. Using purposive sampling, 14 male nurses working in ICUs were recruited from multiple tertiary hospitals across four provinces in China. Semi-structured in-depth interviews were conducted between June and July 2026, and the data were analyzed using Van Manen’s six-step phenomenological analysis method.

**Results:**

Findings revealed a dynamic process underlying male ICU nurses’ experiences of decent work. Participants constructed a sense of decent work through institutional safeguards, social recognition, and professional value. However, this experience was continuously challenged and eroded by high-pressure working conditions, gender stereotypes, and insufficient respect. Despite these challenges, male ICU nurses pursued more decent work through expectations for career development, supportive working conditions, and greater social recognition.

**Conclusion:**

Hospitals and nursing managers should pay attention to the unique challenges faced by male ICU nurses, including professional identity conflicts and insufficient social recognition arising from high-intensity work environments and culturally rooted masculine role expectations within the Chinese context. Targeted efforts should be made to strengthen professional recognition and provide diverse career development pathways to promote the sustainable development of the male nursing workforce.

## 1 Introduction

The global nursing shortage is a pressing global public health challenge that must be addressed. It is worth noting that in recent years, the proportion of men among the global nursing workforce has been gradually increasing (1). Previous studies have suggested that male nurses possess certain relative advantages, including physical strength, mental resilience, and adaptability in clinical settings (2–4). They have become an indispensable part of the nursing team; as a result, male nurses are often employed in critical care units such as ICUs, emergency departments, and operating rooms. These positions are physically demanding and place high demands on nurses’ physical stamina, technical skills, and psychological resilience (5). However, due to factors such as low professional prestige and a lack of professional dignity, male nurses experience significant psychological stress and a marked increase in their willingness to leave their jobs; among male nurses in China, 71.6% express a moderate to high willingness to leave (6). High turnover rates have led to a younger workforce among male nurses, undermining team stability and hindering the development of nursing and improvements in care quality. Furthermore, the proportion of male nurses in China is only about 3.5 percent (7). Well below the global average of 15% (1). These figures not only reflect the shortage of male nurses in China but also reveal the complex lived realities faced by male nurses within the Chinese nursing profession, particularly in high-intensity settings such as the ICU. Decent work perception provides a critical lens for understanding these professional experiences. Originating from the International Labour Organization’s (ILO) Decent Work Agenda, the concept of decent work refers to productive work undertaken under conditions of freedom, equity, security, and human dignity (8). At the individual level, the perception of decent work is defined as an individual’s subjective perception of whether they have obtained or enjoy decent work, and is shaped by their views across five key areas: job security, fair compensation, adequate rest, health insurance, and alignment with personal values (9). Duffy et al. (10) proposed PWT as an empirically testable framework that examines how structural barriers—such as poverty, discrimination, and marginalization—shape individuals’ access to decent work. This framework is particularly relevant to the ICU setting, where male nurses work in high-stress, resource-constrained environments that may threaten their physical safety, psychological well-being, and professional self-worth. PWT thus offers a lens to examine how structural supports shape male ICU nurses’ experiences of decent work. Research (11) indicates that a sense of decent work significantly enhances engagement, thereby reducing nurses’ intention to leave; it does so by enhancing perceived insider status and psychological well-being, which in turn improves nurses’ job performance(12) and positively influences their humanistic practice competencies (13). However, domestic research(14) shows that male nurses in China score lower on the “dignified labor” scale than female nurses, which may be related to the fact that male nurses are mostly assigned to high-intensity, high-risk departments such as ICUs, emergency departments, and operating rooms. At the same time, within the Chinese cultural context, traditional gender role expectations are often encapsulated in the classical notion of “Qian’s vigor and Kun’s compliance” from the I Ching (Book of Changes), which associates masculinity with strength, dominance, and responsibility, while linking femininity with gentleness, supportiveness, and caregiving roles. Under the combined influence of China’s cultural emphasis on “Qian’s vigor and Kun’s compliance,” a professional environment where male nurses lack a strong sense of professional identity, and high-intensity working conditions, male nurses generally have a low perception of decent work and face numerous practical challenges in their career development (15, 16). This suggests that managers should pay special attention to this specific group of male nurses. However, previous studies on decent work among nurses have primarily relied on quantitative surveys. Regarding qualitative studies on decent work, Xue et al. (15) explored the understanding of decent work among general registered nurses, Liu et al. (17) identified themes such as work rewards, value recognition, organizational support, and self-worth realization among specialist nurses, while Abbasi et al. (18) analyzed related threats from the perspective of professional dignity. However, the decent work experiences of male ICU nurses have not been sufficiently explored.

Therefore, guided by the Psychology of Working Theory and adopting an interpretative phenomenological approach, this study aimed to explore the lived experiences and meanings of decent work perception among male ICU nurses in China, thereby deepening the understanding of how male ICU nurses perceive and construct decent work within high-intensity clinical contexts. The findings may provide insights for nursing administrators to develop supportive strategies for sustaining the male nursing workforce.

## 2 Subjects and methods

### 2.1 Study subjects

Using purposive sampling combined with the principle of maximum diversity, registered male nurses were recruited from the ICUs of five Grade A Level III hospitals in Jiangxi, Guangdong, Hunan, and Shandong between June and July 2026. Participants were purposefully selected based on characteristics such as gender (limited to males), age, educational background, years of experience, professional title, marital status, and employment status to ensure a diverse sample and enrich the presentation of professional experiences. Inclusion criteria: (1) Registered male nurses; (2) At least 1 year of specialized ICU experience; (3) Currently assigned to the ICU and routinely participating in clinical shifts. Exclusion criteria: (1) Individuals who had experienced a major occupational trauma event within the past 3 months and were assessed by the researcher as unsuitable for participation in interviews; (2) Individuals on scheduled leave, long-term sick leave, or away for professional training who had not been on regular ICU shifts within the past month; (3) Individuals who had already participated in qualitative research related to the professional experiences of ICU nurses within the past 6 months. Recruitment Process: The researcher, with 4 years of clinical experience in the ICU, identified and recruited potential participants through their existing networks of former classmates, colleagues, and professional contacts in the field. Participants were already known to the interviewer prior to the commencement of the study, and sent recruitment information individually via WeChat, verifying eligibility and exclusion criteria on a case-by-case basis. A total of 18 potential participants were approached; four declined participation due to time constraints or personal reasons, and the remaining 14 were enrolled after providing electronic informed consent. The sample size for this study was determined based on the principle of data saturation. Saturation was operationalized as inductive thematic saturation (19), focusing on the emergence of new codes/themes and informational redundancy. Data collection and analysis proceeded iteratively. After each interview, two researchers independently coded the transcripts using NVivo 12, and the codebook was iteratively updated to track the emergence of new codes (20). By the 12th interview no new themes emerged and existing codes began to recur; two further interviews confirmed this. The research group jointly reviewed the codebook and determined that saturation had been reached. A total of 14 participants were interviewed, all of whom voluntarily participated in this study. All participants voluntarily participated in this study. The participants’ demographic information is presented in Table 1.

**Table 1:** Demographic information of interview participants (*n* = 14).

Number	Age (years)	Marital status	Highest level of education	Years of service	Professional title	Employment status	Region	DWPS score
N1	28	Unmarried	Undergraduate	5	Nurse Assistant	Contract-based	Nanchang, Jiangxi	63
N2	28	Unmarried	Undergraduate	6	Nurse Assistant	Contract-based	Nanchang, Jiangxi	52
N3	27	Unmarried	Undergraduate	4	Nurse Assistant	Contract-based	Nanchang, Jiangxi	35
N4	28	Unmarried	Undergraduate	5	Nurse Assistant	Contract-based	Nanchang, Jiangxi	59
N5	28	Married	Junior College	6	Nurse Assistant	Contract-based	Xinyu, Jiangxi	58
N6	28	Unmarried	Undergraduate	6	Nurse Assistant	Contract-based	Guangzhou, Guangdong	48
N7	26	Unmarried	Undergraduate	5	Nurse Assistant	Contract-based	Nanchang, Jiangxi	59
N8	28	Unmarried	Junior College	5	Nurse Assistant	Contract-based	Nanchang, Jiangxi	61
N9	33	Unmarried	Undergraduate	13	Senior Nurse Practitioner	Permanent establishment position	Nanchang, Jiangxi	69
N10	29	Married	Undergraduate	5	Nurse Assistant	Permanent establishment position	Liaocheng, Shandong	65
N11	29	Married	Undergraduate	9	Nurse Assistant	Contract-based	Changsha, Hunan	61
N12	33	Married	Undergraduate	12	Senior Nurse Practitioner	Permanent establishment position	Changsha, Hunan	55
N13	30	Married	Undergraduate	9	Nurse Assistant	Contract-based	Nanchang, Jiangxi	68
N14	28	Married	Undergraduate	5	Nurse Assistant	Contract-based	Nanchang, Jiangxi	68

The DWPS score refers to the Decent Work Perception Scale score. This scale consists of 5 dimensions and 16 items, with a total score ranging from 16 to 80; a higher score indicates a stronger sense of decent work.

### 2.2 Research methods

#### 2.2.1 Research methods and philosophical foundations

This study was reported according to the Consolidated Criteria for Reporting Qualitative Research (COREQ) checklist, which consists of 32 items for improving transparency and rigor in qualitative research reporting (21). The completed COREQ checklist is provided as Supplementary File S1. This study is grounded in the existential phenomenology advocated by Heidegger (22) and employs hermeneutic phenomenology as its research method. Unlike Husserl’s descriptive phenomenology, which brackets preconceptions to describe the essential structures of experience, Heidegger’s existential phenomenology situates human experience within social, cultural, and relational contexts. While Gadamer emphasizes the interpreter’s role and the historical nature of understanding, Heidegger focuses on how individuals concretely interpret their existence within their particular lifeworlds (23). Since the sense of decent work is not an isolated psychological state but arises within the process of interpersonal interaction—and its experience is deeply influenced by various contextual factors such as interpersonal relationships and organizational atmosphere—hermeneutic phenomenology is well-suited for its exploration. This study further employs van Manen’s (23) six-step analytical method for data analysis. This approach aligns with interpretive phenomenological research and emphasizes a holistic understanding of lived experience and the interpretation of meaning, thereby helping to reveal the layered constructional characteristics of male ICU nurses’ sense of decent work and the intrinsic connections among the themes.

#### 2.2.2 Developing the interview guide

A preliminary draft of the interview guide was developed based on clinical experience and a literature review. Two eligible ICU male nurses participated in pilot interviews to assess the clarity of the questions and the feasibility of the interview process. Based on their feedback, the research team refined the guide. The question “What factors contribute to your perception of decent work?” was revised to “Which scenarios make you feel a sense of decent work?”—redirecting the focus from abstract factors to concrete workplace experiences. Additionally, the probe “Could you give an example?” was added after each core question to encourage participants to elaborate with specific clinical examples. After discussion and revision, the final interview guide was established as follows: (1) How do you interpret the term “decent work”? (2) How do you feel about your current job? (3) In what situations do you feel a sense of decent work? Can you provide examples? (4) Conversely, in what situations do you not feel a sense of decent work? Please provide examples. (5) What measures would you like to see implemented to enhance male nurses’ sense of decent work?

#### 2.2.3 Data collection methods and quality control

The researchers made initial contact with study participants via WeChat, established a relationship of trust with them, explained the purpose and significance of the study, and asked if they were willing to participate. Once their willingness to participate was confirmed, both parties agreed on the time and format of the interview, and an electronic informed consent form was sent via WeChat. The interview began after the participant gave their consent. One-on-one interviews with participants were conducted in virtual rooms on Tencent Meeting to ensure a quiet and comfortable environment free from disturbances by unrelated individuals, thereby protecting participants’ privacy. During the interviews, the Tencent Meeting recording function was used to record the sessions, while the researcher simultaneously took notes to document nonverbal cues such as facial expressions and body language. In principle, each interview was limited to 30–60 min, with adjustments made as appropriate based on the interviewee’s willingness to speak and the level of information saturation. Each participant was interviewed once. No repeat interviews were conducted. Research (24) has confirmed that online interviews can serve as an alternative to in-person interviews. Furthermore, the physical separation provided by online interviews reduces social desirability bias and evaluation pressure, allowing participants to feel safer when discussing sensitive topics and to disclose more information. After each interview, a reflection journal was written promptly, and the transcribed materials were organized and returned to the interviewee electronically for confirmation.

#### 2.2.4 Assessment tool for perceptions of decent work

Prior to the formal interviews, participants in this study were asked to complete the Decent Work Perception Scale (DWPS) (25). The scale consists of 16 items across 5 dimensions: 4 items on work rewards, and 3 items each on job position, work environment, career development, and professional recognition. A 5-point Likert scale was used, ranging from “Strongly Disagree” to “Strongly Agree” and scored from 1 to 5, respectively. The total score ranges from 16 to 80, with higher scores indicating a stronger perception of decent work. The scale’s Cronbach’s α coefficient is 0.745. The purpose of introducing the Decent Work Perception Scale (DWPS) was to describe the basic characteristics of the participants, serving as background information directly related to the research topic, thereby enriching readers’ understanding of the sample and providing a reference for assessing the transferability of the findings. The role of the scale is not to provide quantifiable analytical evidence but to serve as an auxiliary tool. It was not used as an inclusion or exclusion criterion. Furthermore, the scale scores were not referenced during data analysis, coding, or theme development. All qualitative themes in this study are derived entirely from the participants’ interview narratives.

#### 2.2.5 Data analysis methods

Within 24 h of the completion of each interview, the researcher transcribed the audio recordings verbatim and proofread and supplemented the transcripts based on notes; data organization and analysis were conducted simultaneously. This study adopted an interpretive phenomenological approach aimed at describing the essential experiences of male ICU nurses regarding their sense of dignified labor, using van Manen’s analytical method to analyze the interview data (23). Data management and coding were assisted using NVivo 12 software. The specific steps are as follows. The data analysis steps included: (1) Gaining an overall sense of the textual data. Repeatedly reading the transcripts to form a comprehensive understanding of male ICU nurses’ perceived experiences of dignified labor; (2) Extracting units of meaning. Focusing on the research objectives, meaningful statements related to the sense of dignified labor were extracted from the interview transcripts; for example, “Doing this means earning an income commensurate with one’s efforts—to put it plainly, coming to work is about making money. Only when wages and benefits improve will this job feel decent.” Into “decent work grounded in salary security”; (3) Grouping similar units of meaning. Compare and categorize the extracted units of meaning; for example, integrate “decent work grounded in salary security” and “decent work grounded in safety support” to form the meaning cluster “decent work grounded in institutional safeguards”; (4) Conduct thematic analysis. Conduct an in-depth analysis of the essential connotations of each semantic cluster to reveal the logic behind the construction of male ICU nurses’ sense of dignified labor and the mechanisms that erode it; (5) Establish themes and clarify the connections between them. Further integrate the analysis results to establish core themes such as the “security–recognition–value” hierarchical structure and the “overdraft–erosion–damage” erosion process, and elucidate the intrinsic connections between these themes. To ensure analytic transparency, Supplementary File S2 tracks the progression from raw quotes to themes using illustrative examples. Themes were inductively derived from data, not preset by PWT. PWT was then applied as an interpretive lens to examine how structural, occupational, social, and cultural contexts shaped participants’ decent work experiences. (6) For member checking, preliminary findings were shared with all 14 participants to confirm they reflected their actual experiences. All confirmed the themes captured their experiences, validating the results. To ensure the credibility of the findings, two researchers trained in qualitative research independently coded the interview transcripts. Their coding results were compared, and disagreements mainly concerned the identification of meaning units, code labeling, and the grouping of codes into categories. These disagreements were discussed with a third researcher by referring to the original transcripts and contextual information until consensus was reached. This study adopted an iterative strategy in which data collection and preliminary analysis proceeded simultaneously. Two researchers independently read the interview transcripts and conducted preliminary coding; after completing each interview, they compared their codes and organized the themes. By the 14th interview, no new units of meaning or thematic categories emerged, and the codes already extracted began to recur and show signs of information redundancy in subsequent interviews, leading to the conclusion that the data had reached saturation. Following joint confirmation by the research team, data collection was halted, and a total of 14 interviewees were ultimately included in the study.

#### 2.2.6 Ethical considerations

This study has been reviewed and approved by the Medical Ethics Committee of Hunan Normal University, with approval number 2026565. During recruitment, participants were informed of the study purpose, procedures, confidentiality measures, and their right to withdraw at any time, and they signed informed consent form. Interviews were conducted via Tencent Meeting in a private setting. No one else was present during the interviews. Audio recordings and transcripts were securely stored on a password-protected device and were accessible only to the research group. All data were anonymized, and recordings and related materials will be deleted after 5 years.

#### 2.2.7 Ensuring rigor

Rigor in qualitative research is not an evaluation criterion applied only after the study is completed, but rather a core element that runs throughout the entire research process and allows for self-correction (26). In addition to describing the research process as thoroughly and transparently as possible, this study also promoted rigor through the following measures:

1. The researcher is currently a postgraduate nursing student and previously worked as a male ICU nurse for 4 years. The researcher’s interest in this topic arose from his clinical experience, during which he observed and experienced how work pressure, professional recognition, and gender stereotypes influenced the occupational experiences of male nurses in the ICU. As an ICU nurse like the research participants, was an “insider” in terms of role. This identity facilitates an understanding of the ICU work environment, fosters empathy with the research participants, and allows for a deep grasp of their professional experiences. However, the “insider” status may also introduce preconceptions and reduce sensitivity to the data, and may also influence participants’ expression as well as the researcher’s interpretation of the data. Additionally, participants were recruited through personal networks, and the researcher had pre-existing relationships with some of them, which could create power imbalances and social desirability bias. To mitigate this, the researcher explicitly stated at the start of each interview that participation was voluntary, there were no right or wrong answers, all responses were equally valued, and that participants’ employment status would not be affected by their responses. To mitigate this bias, a reflective journal was maintained throughout the study, continuously documenting the researcher’s inherent perceptions, impressions during interviews, and interpretive approaches, enabling the timely identification and setting aside of subjective assumptions to ensure the objectivity and rigor of data analysis.
2. Credibility: A summary of the preliminary thematic findings was shared with all 14 participants via WeChat. Participants were given 1 week to review the findings and were invited to confirm their accuracy, suggest revisions, or add further perspectives. All 14 responded and confirmed that the findings reflected their intended meanings; no revisions were requested.
3. Transferability: Thick descriptions were provided to facilitate judgments regarding transferability. The study described the research context, ICU working environment, participant characteristics, and organizational settings in detail. Participants were recruited from ICUs of five tertiary hospitals across four provinces in China, with variation in age, educational background, professional title, and years of ICU experience. These contextual descriptions allow readers to evaluate the applicability of the findings to similar clinical settings.
4. Dependability: An audit trail was maintained throughout the research process to enhance dependability. The research team preserved original interview recordings, verbatim transcripts, NVivo12 coding files, coding records, analytical memos, theme development processes, and team discussion notes. These materials documented the progression from raw data to final themes and ensured transparency and traceability of the analytic process.
5. Confirmability: The reflexive journal was maintained throughout the research process. For example, when N1 said he did not go out of his way to correct patients who mistook him for a doctor, I paused. In my own past practice, I would immediately correct such misidentifications and would feel quite angry. His calm response was not what I expected and made me realize that I had unconsciously projected my own experience onto his. I reminded myself to set aside my own reactions in subsequent interviews and focus on listening to participants‘own perspectives.

## 3 Results

In this study, participants’ DWPS scores ranged from 35 to 69. Reporting this range was intended to provide additional contextual information regarding the variation in participants’ perceptions of the core phenomenon examined in this study, namely decent work, indicating that the included participants did not demonstrate a concentrated or uniform level of perceived decent work. Meanwhile, the interview data further revealed diverse experiences and meaning constructions related to decent work among participants. It should be emphasized that participants were not grouped according to their DWPS scores, and no case-level correspondence, comparison, or analysis was conducted between individual scale scores and specific interview data. Therefore, this study does not make further inferences regarding the relationship between variations in DWPS scores and specific themes or individual experiences. All themes were generated exclusively from the phenomenological analysis of the interview data. Through data analysis, this study identified three main themes and eight subthemes. From the perspective of the Psychology of Working Theory (PWT) (10), male ICU nurses’sense of decent work unfolds within a dynamic process of “construction—erosion—pursuit”: individuals actively construct a sense of decent work through institutional safeguards, social recognition, and professional values, reflecting the proactive process within PWT of pursuing decent work to meet needs for survival, safety, social connection, and self-determination; However, under structural barriers such as high-intensity labor, gendered labeling, and demeaning interactions, this sense of decent work is continuously eroded; at the same time, participants consistently pursue more decent work conditions, resulting in a complex perception characterized by an interplay of dynamism, fragility, and incompleteness (Figure 1).

**Figure 1:**
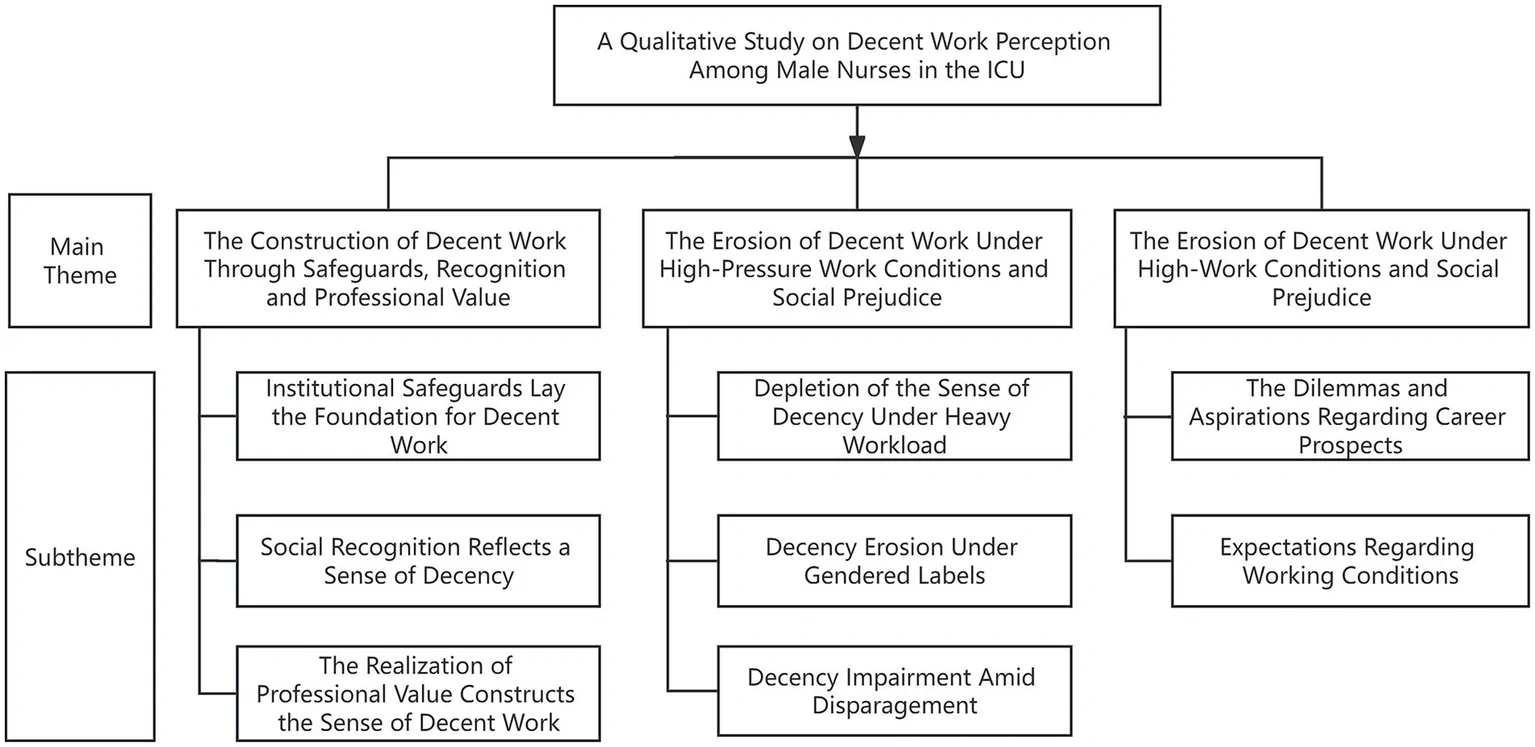
Thematic framework for male ICU nurses’ experiences of decent work.

### 3.1 Theme 1: the construction of decent work through safeguards, recognition and professional value

Male ICU nurses’perception of decent work is gradually constructed through the interaction of institutional safeguards, social recognition, and professional value realization. Institutional safeguards provide the fundamental conditions for decent work, social recognition strengthens their professional identity, and the realization of professional value further enriches the meaning of their work. Together, these dimensions represent the initial construction of decent work among male ICU nurses.

#### 3.1.1 Institutional safeguards lay the foundation for decent work

Institutional safeguards lay the foundation for decent work, primarily by ensuring that jobs provide workers with a safe working environment, reasonable compensation, and adequate rest time. These elements constitute the most fundamental and core pillars of decent work.

N11: “This line of work isn’t exactly safe. Especially in the ICU—if a patient punches you, you can’t fight back, and you can’t call the police either” (wry smile). N5: “There are family members who don’t understand or support our work, and they might verbally abuse us—or even physically assault us.” N12: “The main issue is that the shift handover process in our department takes too long. If we have a lot of patients, by the time the entire handover is complete, we end up working about half an hour of overtime before we can finally clock out. There’s really nothing we can do about that.” N4: “I think not being able to take time off for night shifts or during holidays and festivals really affects me quite a bit; most of my negative emotions stem from that.” N10: “We work to make a living. If the take-home pay is low, I might be more likely to consider quitting. Other situations can actually be overcome—even being tired is fine—but it’s all for those meager earnings” (slight curl of the lips, self-deprecating tone). N3: “Decent work is tied to salary, isn’t it? When I tell friends I’m a male nurse, their first reaction is, ‘How can you do that?’ But if you say, ‘I make this much a month,’ and the number is high enough, they’re surprised. That’s what decent work means in the conventional sense.”

#### 3.1.2 Social recognition reflects a sense of decency

Social recognition reflects decent work by enabling male nurses to receive positive feedback in clinical practice. This recognition helps them develop a sense of professional achievement. At the same time, support from the professional environment—such as mutual assistance among colleagues and team harmony—further strengthens their professional identity. Together, these two factors form the relational foundation of decent work, enabling them to perceive the value of their labor and their sense of decent work.

N4: “I think the greatest recognition comes from patients. If a patient says you’ve taken good care of them and is satisfied with your actions and work, I feel that’s worth more than anything.” N14: “Recognition from family members is the best reward for our work. When family members thank us or write thank-you letters, it makes this job feel quite rewarding” (eyes brightening with a warm smile). N3: “I’m the one who carries out the tasks, while the doctors make the decisions. When I execute my duties well and receive positive feedback, that’s also a source of motivation for me.” N12: “I’d say it’s recognition from my supervisor. When they’re willing to assign new staff to you to train, it probably gives you… well, a sense of accomplishment.” N9: “It’s probably recognition from my supervisor—affirmation of my work. It’s the same psychological feeling as when a child is praised by a parent or teacher.” N5: “I’m from the north and don’t have any relatives or friends here, but working in the ICU is all about teamwork. It’s a warm environment—we all work hard together, chat and laugh, and feel pretty relaxed. That’s what I like most about it.”

#### 3.1.3 The realization of professional value constructs the sense of decent work

The realization of professional value for male nurses stems not only from the full exercise of their professional abilities in daily nursing work, but also from the sense of self-fulfillment derived from critical events such as successfully resuscitating patients. It is further exemplified by the profound elevation of the meaning of their work when personal value and social value are unified.

N11: “You have to be professional enough for others to respect you. If all you can do is give injections and dispense medication, what’s the difference between a tertiary hospital and a clinic? You have to be able to explain the condition clearly—what the illness is, how to treat it, and what to watch out for at home. If you can provide value to patients, they’ll naturally see your value.” N5: “We nurses are on duty in the ward, so we’re the first to know when a patient’s condition changes. For example, if their blood oxygen level suddenly drops or their lips or complexion becomes cyanotic, we immediately assess the situation and suction their airways, and the patient regains consciousness. This is the most basic aspect of professional competence: knowing how to observe, make judgments, and intervene in time.” N7: “Assisting doctors with procedures—like endotracheal intubation or tracheostomies—allows us to make a tangible difference.” N6: “For example, when a young patient suffers massive bleeding from a car accident and goes into cardiac arrest, and you perform CPR to save their life—and later when the patient was discharged, he later sent me a thank-you banner—that moment feels truly wonderful” (speaks more slowly, eyes brightening). N10: “When we admit critically ill patients and immediately begin resuscitation—that kind of scene feels quite sacred, and it instills a very strong sense of mission.” N3: “When you complete your work tasks, you feel a sense of accomplishment—that’s personal value, and these are all part of what makes work decent. Social value comes from contributing to society; look at police officers who maintain law and order and doctors who save lives and treat the injured—these are all contributions to society.”

### 3.2 Theme 2: the erosion of decent work under high-pressure work conditions and social prejudice

Although male ICU nurses actively construct a sense of decent work through institutional safeguards, social recognition, and professional value realization, this sense of decency remains dynamic and vulnerable, as it can be continuously challenged and eroded in clinical practice.: first, by high-pressure environments, such as the mental strain of caring for critically ill patients and the physical and mental toll of high-intensity work; second, by social prejudice, such as the professional challenges arising from gender stereotypes; and third, by the loss of decent work resulting from a lack of respect.

#### 3.2.1 Depletion of the sense of decency under heavy workload

The erosion of decent work is manifested through several key aspects.: In the high-pressure environment of the ICU, male nurses face dual pressures—both physical and mental—leading to a gradual erosion of their sense of decent work.

N3: “There are some emotions or thoughts you just can’t put into words. People need not only to be listened to, but also to speak their minds. Yet most of the time, we’re just playing the role of listeners, always trying to solve our patients’ problems. After listening so much, we end up becoming the patients ourselves. Over time, isn’t that just a mental health issue?” (looks down and sighs) N7: “I used to have trouble sleeping after night shifts, and I couldn’t sleep the night before a day shift either. We have a lot of night shifts—over 100 a year!” N10: “The ICU is physically demanding; physical exhaustion and lower back strain are definitely common. After working here for a few years, you’ll have some wear and tear on various parts of your body. Then there’s problems such as perceived liver problems related to staying up late—that happens too.” N10 (married): “There are a lot of emergencies at work, so you have to be on call at all times—ready to respond immediately, no matter if it’s a holiday or not. I guess I haven’t been able to spend as much time with my family as I’d like.” N8: “There are so many alarms in the ICU—from ventilators, monitors, and all kinds of medical equipment. After hearing them so often, I’ve become extremely sensitive to those sounds. Sometimes, even when there’s no actual alarm, it feels like I’m hearing a sound in my head—almost like auditory hallucinations.” N11: “For a while, I grew to dislike this profession—work gave me a kind of PTSD. Every night I’d have nightmares, either about resuscitation attempts or the beeping sounds of machines. Just thinking about going to work made me anxious. Plus, when I first started, I’d inevitably make mistakes, and after my shift, I’d get calls and be tagged in group chats. Back then, I didn’t want to go to work at all—I was just full of resentment” (furrowed brows).

#### 3.2.2 Decency erosion under gendered labels

As society has evolved, more men have entered the nursing field due to their physical strength and psychological resilience. However, in clinical practice, they still face prejudice from patients, family members, and society at large, and their decent work conditions are gradually eroded. N13: “When I wear that uniform—and as a man—people assume I’m a doctor, so I just tell them I’m a male nurse. Some react strongly, saying, ‘Men can be nurses too?!?’ Most ordinary people don’t have much exposure to male nurses” (looks resigned). N1: “Some family members entering the ICU might ask, based on my gender, if I’m a doctor. If patients think we’re doctors, they might be a bit more compliant. I don’t go out of my way to correct that.” “N8: “Some female patients feel it’s a bit awkward to have a male nurse present during certain procedures. For things like sponging, ECGs, or catheterization, female patients value their privacy, so this can cause a bit of discomfort.” N3: “Besides, it’s just a job—why distinguish between men and women? There’s already a bit of gender conflict within the profession. For the same work, why is it assumed that men should do more and give way to women? Society’s recognition of nurses—at least in China—isn’t that high, and that’s hard to change. It’s like trying to cut down a big tree and plant another one just as big—how many years would that take?” N6 (unmarried): “To be honest, male nurses are still at a disadvantage when it comes to finding a partner. We’re competing with doctors, whose salaries and benefits are generally much higher than those of male nurses. They have much more leverage in the dating market—we’re essentially at a disadvantage.” N12: “Sometimes, when performing a venipuncture, it’s perfectly normal for a female nurse to fail. But if a male nurse fails, people are quick to label him—basically assuming that men aren’t well-suited for this kind of work, and they jump to that conclusion right away.”

#### 3.2.3 Decency impairment amid disparagement

In their clinical work, male nurses often face skepticism and complaints from patients or their families, and are even treated as if they were waitstaff. This demotion makes them feel humiliated and as though they have lost all face, thereby damaging their sense of decent work. N13: “Sometimes we encounter patients who don’t understand. They’ll make demands that they think are reasonable but we don’t. For example, a postoperative patient might feel discomfort in their throat after being extubated and want a drink of water. We don’t give water that early after surgery. When we explain this to them, they think that since they’re awake, they’re fine and absolutely must have a drink. If we refuse, they just can’t accept it, and then they turn around and file a complaint against you, holding a grudge.” N7: “When communicating with family members, some can be quite difficult to deal with. For example, if a patient has a fever, they’ll blame us; when we cool them down, they’ll complain it’s too cold and ask why we didn’t cover them with a blanket—there are so many incidents like this. Some family members even yell at you.” N4: “It might happen a little less often in the ICU, but on the general wards, we’re sometimes treated like waitstaff. In my five years on the job, I’ve been bitten, scratched, kicked, and spat on. I feel deeply disrespected, and my self-esteem has been crushed” (voice low, expression despondent). N9: “Our profession isn’t exactly respected, is it? Nurses don’t seem to have a very high status. Family members only listen to the doctors—one word from a doctor carries more weight than ten from a nurse.” N11: “In our country, the general public still tends to view nursing as a service industry—a non-technical job. Although there are some highly specialized procedures, my impression is that most people treat you as if you’re in a service role; for example, they call you a ‘waiter.’”

### 3.3 Pursuit of more decent work amid expectations

Although male nurses continue to face challenges in their current work environment, they still hold expectations for a better future. They hope to be more visible and receive greater recognition from society. Through various efforts to improve their working conditions and professional experiences, they continue to pursue a more decent and valued work life.

#### 3.3.1 The dilemmas and aspirations regarding career prospects

Male nurses hold mixed perceptions of their career prospects. They are aware of the relatively clear promotion pathways in nursing. However, they also face limitations in career advancement and a shortage of leadership positions. Therefore, they often rely on personal efforts to gradually improve their career development.

N3: “I’ll develop slowly and study new technologies diligently. I hope to move up, but I don’t hold out much hope for reaching management—I don’t have any standout qualities to compete with others. If I’m still in this position in middle age without having climbed to management, it won’t be dignified. It takes a lot of courage to tell people I’m a male nurse when I’m almost 40. When I think about spending the next 20 years as a frontline nurse, I don’t even want to do this job anymore”(sighed softly). N4: “I’ll do my best to advance my professional title and improve my expertise. It’s hard to plan for the future—will I still be capable of handling critical care in the ICU ten years from now? As women get older, they might be reassigned to less demanding departments, but I rarely hear of men being transferred to relatively less demanding departments like outpatient clinics, geriatric wards, or VIP wards.” N11: “I think I still need to read more; I might pursue a master’s degree. Whether it’s for future professional title promotions or if I really want to advance and become a head nurse, our hospital currently doesn’t even allow those without a master’s degree to run for the position.” N9: “My plan is to focus more on learning and participating in various training programs to improve myself. For example, attending off-site training, pursuing specialized nursing certifications, and earning more professional credentials to develop expertise in a specific area—such as intravenous therapy, nutrition, or rehabilitation nursing.”

#### 3.3.2 Expectations regarding working conditions

Expectations regarding working conditions mainly reflect male nurses’ desire for improved salary and greater professional recognition. They also hope to receive more attention and support from hospitals, society, and the government. These supports can further enhance their perception of decent work. N9: “I hope my income will be more substantial, that the nurse-to-bed ratio will meet reasonable standards, and that I’ll have more say in decision-making.” N14: “I think the government should raise salaries and benefits for healthcare workers. Since the implementation of the current healthcare insurance policy, nurses’ wages have generally declined; many have to take on side jobs just to make ends meet because their primary income alone is still too low. If salaries and benefits continue to improve, there will definitely be fewer people leaving the profession.” N8: “I hope there will be more care and support for male nurses. Look, female colleagues even get the day off on International Women’s Day.” N6: “I think hospitals and society should organize more social events, including sharing sessions like this one, where we can pass on what we’ve learned to others. That might help improve the nursing profession as a whole.” N10: “Male nurses should be more visible in public so that the public recognizes their importance. Also, in terms of pay and career development, there should be a greater focus on them, because after all, men don’t get pregnant or give birth, so we’re more convenient for employers to deploy.”

## 4 Discussion

This study aimed to explore the lived experiences of decent work perception among male ICU nurses in China. By analyzing the experiences of male nurses in the ICU regarding their sense of decent work, we find that this sense of decent work first stems from the protection of individual rights, is then affirmed and reinforced through social recognition, and reaches its peak through the realization of personal value and the acknowledgment of social contributions. However, high-pressure work environments and social prejudices continue to erode this sense of decent work, prompting male nurses to envision their future amidst a balance of perseverance and hope. By fully understanding the composition, erosion, and expectations surrounding the sense of dignified labor, we can gain preliminary insight into its formation mechanisms and, in turn, explore strategies to enhance this sense of dignified labor. From the perspective of the Psychology of Working Theory (PWT), these findings suggest that decent work among male ICU nurses is shaped by the fulfillment and disruption of three fundamental needs: survival, social connection, and self-determination.

### 4.1 Building a support system to promote the construction of decent work

The interviews revealed that the “security–recognition–value” hierarchical structure of male ICU nurses’ sense of decent work reflects their step-by-step psychological aspirations during the work process: “seeking basic security—gaining social recognition—realizing spiritual pursuits.” Fundamentally, this resembles the hierarchical structure of needs in Maslow’s (27) Hierarchy of Needs theory. This similarity is reflected in the fact that basic needs may lay the foundation for individuals to pursue higher-level psychological fulfillment. However, unlike the stepwise progression postulated by Maslow’s hierarchy of needs, the three dimensions identified in this study tend to be interwoven, mutually reinforcing, and characterized by recursiveness and context-dependence. This layered mechanism can be understood from three perspectives: First, under traditional notions of gender roles within the family, men are expected to serve as the family’s economic breadwinners, making them more sensitive to instrumental safeguards such as compensation, benefits, and labor protection; Second, many male nurses enter the nursing profession through reassignment or due to employment pressures rather than by active choice; consequently, they have a relatively low sense of professional self-identity and rely more heavily on external evaluations to validate the rationality of their career choice; third, some male nurses possess strong altruistic tendencies and a sense of self-efficacy, and tend to realize their self-worth through the treatment of critically ill patients.

Consistent with the dimensions sorted out by Hult et al. (28) institutional guarantees, workplace recognition, and professional values constitute the core pillars of decent work. Xue et al. (15) study on decent work for nurses in the Chinese context similarly indicates that institutional safeguards, external recognition, and professional value are core components of nurses’ sense of decent work. These findings can also be understood through PWT. Institutional safeguards mainly reflect survival needs, social recognition reflects the need for connection, and professional value reflects self-determination through meaningful and competent work. The “layered construct” identified in this study resonates with the aforementioned research but further reveals the unique characteristics of male nurses arising from gender role expectations—their prioritization of basic safeguards is not merely a “material need,” but rather an adaptive strategy resulting from the clash between masculine cultural scripts and the nature of the nursing profession. Furthermore, the high-intensity, high-risk, and high-workload nature of ICU practice (29) amplifies the urgency of needs at all levels, making the perception of dignified labor more prone to the vulnerability of “an unstable foundation leading to instability at higher levels.” The hierarchical framework of this study suggests that medical institutions can significantly enhance nurses’ perception of decent work (30) by improving organizational support systems and focusing on both instrumental support (salary and benefits, working conditions, and rights protection) and emotional support. This should be implemented collaboratively and in a tiered manner by hospitals and head nurses. These findings may offer guidance for developing a multi-level strategy to support decent work among nurses: at the level of safety and security, hospitals could further improve protection and remuneration systems and optimize resource allocation to ensure adequate rest, while head nurses, through reasonable shift scheduling and timely motivational feedback, may help lay the foundation for decent work (31–33); at the level of social recognition, hospitals might promote professional image and improve reward mechanisms, while head nurses could focus on conveying positive feedback from patients, families, and the team, which may help create a supportive atmosphere and enhance professional accomplishment; at the level of professional value, hospitals could explore establishing clearer career advancement pathways and provide opportunities for further training and showcasing (18), while head nurses might support nurses’ participation in critical care, specialized training, and teaching practice, which may, to some extent, facilitate the translation of professional competence into professional value.

### 4.2 Creating an environment of respect and support to counteract the erosion of decent work

This study shows that male ICU nurses’sense of decent work is continuously eroded in three ways: high-intensity work leads to the depletion of decent work, gender stereotypes cause the erosion of decent work, and a lack of respect results in the impairment of decent work. Viewed through PWT, these experiences represent barriers to fulfilling basic needs for decent work. High-intensity work threatens survival needs, while gender stereotypes and lack of respect undermine social connection and professional identity. Similar to the findings reported by Ajayi et al. (34) that high-intensity work and external disrespectful behavior can damage nurses’ decent work perceptions. A survey by scholar Wang et al. (35) revealed that male nurses in China generally have a low perception of decent work and pointed out that high-intensity work, workplace disrespect, and the stress of night shifts significantly undermine nurses’ perception of decent work. This study further found that gender stereotypes are a source of erosion unique to male nurses. Lyu et al. (4) described gender as a “double-edged sword” for male nurses: they may face stereotypes, discrimination and role strain, while also gaining advantages related to physical strength, patient preference and career opportunities. These findings suggest that the impact of gender is complex and context-dependent. In the present study, however, the erosive aspects of gender appeared more salient, which may be related to the high-intensity ICU environment and the Chinese cultural context. The traditional Chinese gender concept of “Qian is strong, Kun is submissive” profoundly influences the public’s perception of male nurses. This ideology originates from the Book of Changes’ (I Ching) assertion that “Qian represents strength, and Kun represents submission,” positioning men in the role of being strong and decisive, while women are seen as gentle and supportive (36). Consequently, the public has internalized the perception that “men take the lead as doctors, while women serve as nurses,” leading to instances of misidentification. At the same time, the “saving face” (37) culture in traditional Chinese society further exacerbates the challenges faced by men in the nursing profession. “Face,” as a core concept in Chinese social relations, emphasizes an individual’s social image, dignity, and status in the eyes of others. This cultural pressure places male ICU nurses in a dilemma regarding their identity. Influenced by traditional notions, male nurses are often mistaken for doctors, and in some cases, they choose not to correct this perception. This is both a choice made by practitioners to maintain their own image and a reflection of the psychological conflict male nurses face between societal expectations of male roles and the nature of the nursing profession. It also highlights the fragility and defensiveness of male nurses’ sense of decent work. The findings may inform strategies to resist the erosion of decency: at the level of decency depletion, hospital management could, taking into account the specific characteristics of ICU work, pay further attention to the balance among shift scheduling, nursing staffing, and rest guarantees, which may help alleviate the adverse effects of long-term high-intensity work on male nurses’ professional experience (38); at the issue of gender labeling, within the department, task allocation could be based on competence and downplay gender differences, while in intimate care, adopting a practice of “same-gender care priority combined with informed consent” may help reduce the impact of gender stereotypes, and externally, through publicizing exemplary male nurse stories and conducting community science popularization, these efforts might also provide some reference for gradually improving public perception; at the level of decency impairment, hospital management could explore more comprehensive approaches to doctor-patient communication and conflict resolution (39), reduce double standards arising from gender labeling, and emphasize enhancing nurses’ professional voice and support for those whose rights have been infringed, thereby reducing the potential secondary harm from “passive tolerance.”

### 4.3 Listening to the inner aspirations of the group to support long-term career development

In this study, most male ICU nurses did not express a desire to leave their jobs, but respondents generally reported a strong desire for more care and support. Amid the ongoing erosion of decent work conditions, male ICU nurses, on the one hand, have expectations regarding working conditions—such as compensation, scheduling and rest periods, humanistic care, and a voice in the industry—while, on the other hand, they aspire to career advancement but are constrained by practical challenges such as limited promotion opportunities, a scarcity of management positions, and difficulties in job reassignment for older employees. Studies by Bayer (40) and others have also confirmed that high-intensity shift work, insufficient room for growth, and a lack of organizational support are common professional challenges faced by male nurses across countries; work resources and career development opportunities directly influence their professional attitudes and willingness to remain in the profession, which aligns with the findings of this study. A study by Cai et al. (41) points out that male nurses in China generally exhibit a high propensity to leave their jobs, and that the perception of decent work is a core factor influencing this tendency. Poor working conditions, obstacles to career development, and a lack of organizational support—coupled with practical concerns regarding marriage, relationships, and family—all significantly undermine practitioners’ perception of decent work, which aligns with the demands and challenges identified in this study. These findings may provide a basis for informing long-term career development planning for male nurses: First, promoting more transparent and open promotion to management positions, creating a professional environment that prioritizes competence regardless of gender, and exploring the establishment of diversified career pathways encompassing specialized technical skills, teaching, administrative management, research, and community nursing, may help to divert promotion pressure and expand development opportunities (42, 43); Second, for nurses who are married, have heavy family burdens, or experience declining physical fitness due to aging, exploring the establishment of job rotation or transfer mechanisms may, to some extent, achieve a dynamic balance in workload; Third, addressing marital and relationship difficulties requires multi-party collaboration: at the societal level, public education to eliminate occupational prejudice and enhance professional recognition; at the hospital level, optimizing flexible scheduling and shift adjustment systems, and trade unions organizing cross-industry social activities to safeguard private social time; and at the individual level, strengthening professional confidence and interpersonal skills, and the parallel implementation of these measures may help alleviate obstacles to marriage and relationships such as mismatched work schedules, limited social circles, and occupational misunderstandings. Overall, male ICU nurses’ pursuit of better working conditions and career development reflects their ongoing efforts to restore unmet needs for survival, social connection, and self-determination.

Overall, this study indicates that decent work among male ICU nurses is a dynamic process shaped by the interplay of protection, recognition, and professional value. The findings suggest that improving nurses’ work experiences requires attention not only to organizational support and career development, but also to social perceptions of the nursing profession. From a theoretical perspective, the construction–erosion–pursuit framework provides a contextualized understanding of how decent work is continuously formed and challenged in nursing practice.

### 4.4 Limitations

This study has the following limitations: First, participants were primarily recruited through the researcher’s personal WeChat network. This online recruitment method may introduce selection bias, thereby limiting the diversity of perspectives to some extent. Second, the sample was geographically concentrated, with 8 of the 14 participants hailing from Nanchang, Jiangxi. Consequently, the findings may not fully reflect the experiences of male ICU nurses in other regions of China, which could limit the generalizability of the results. Third, this study relied on one-time interviews, which capture participants’ experiences at a single time point and cannot show how perceptions of decent work evolve over time. Future studies could adopt broader recruitment strategies to include participants from more regions, thereby enhancing sample diversity and improving the generalizability of the findings. Fourth, the DWPS was used to describe participant characteristics, but differences in scores and their influencing factors were not further explored. In addition, combining quantitative measurement with interpretive phenomenology may introduce methodological tension. Future studies could adopt mixed-methods designs to integrate quantitative assessment with lived experiences and further understand how decent work perceptions develop.

## 5 Summary

This study employed an interpretive phenomenological approach combined with work psychology theory to explore the lived experiences of decent work among ICU male nurses in China. The findings indicate that perceptions of decent work are not static but are continuously constructed through institutional support, social recognition, and professional value realization. Meanwhile, these perceptions are persistently challenged by high-intensity work demands and gender-related cultural expectations, while male nurses continue to seek more decent working conditions through perseverance and hope. This study makes three main contributions. First, it reveals the dynamic process of “construction–erosion–pursuit” in ICU male nurses’ experiences of decent work from a phenomenological perspective, extending previous research that has primarily focused on occupational stress, professional identity, and turnover intention among male nurses. Second, it highlights the influence of gender role expectations and face culture within the Chinese cultural context, providing new insights into gendered experiences in the nursing profession. Third, it extends the application of work psychology theory by illustrating how needs for survival, social connection, and self-determination are understood and addressed within gender-sensitive and culturally specific clinical contexts. In practice, nursing managers should improve male nurses’ work experiences through enhanced occupational security, organizational support, and career development opportunities. Nursing education should promote professional identity development and gender role reflection to reduce the impact of stereotypes. Nursing workforce policies should also focus on career pathways and workplace support systems for male nurses to promote workforce stability. This study has several limitations. Network-based recruitment and regional concentration of participants may limit the transferability of the findings, and single-time interviews could not capture changes in perceptions of decent work over time. Future research could adopt longitudinal designs to examine the dynamic evolution of these experiences across career development and changing work environments. Comparative studies across different clinical departments, healthcare institutions, and cultural contexts may further identify contextual variations and inform more targeted policies and management strategies.

## Data Availability

The raw data supporting the conclusions of this article will be made available by the authors, without undue reservation.
