# Supplementary material for: A qualitative study on decent work perception among male nurses in the ICU
Source: Front Public Health. 2026 Oct 16;14:1942420. doi: 10.3389/fpubh.2026.1942420 (PMC13719544; doi:10.3389/fpubh.2026.1942420)
Supplement: Supplementary file 1 [file Table_1.docx]

**COREQ(COnsolidated criteria for REporting Qualitative research)Checklist**

A checklist of items that should be included in reports of qualitative research.You must report the page numberin your manuscript where you consider each of the items listed in this checklist.If you have not included this information,either revise your manuscript accordingly before submitting or note N/A

| **Topic** | **Item No.** | **Guide Questions/Description** | **Reported on Page No.** |
| --- | --- | --- | --- |
| **Domain** **1:Research** **team** **and** **reflexivity** | | | |
| Personal chorocteristics | | | |
| Interviewer/facilitator | 1 | Which author/s conducted the interview or focus group? | Methods, 1.2.2 Data Collection Methods and Quality Control |
| Credentials | 2 | What were the researcher's credentials?Eg.PhD,MD | Methods, 1.2.6 Ensuring Rigor |
| Occupation | 3 | Wha was their occupation at the time of the study? | Methods, 1.2.6 Ensuring Rigor |
| Gender | 4 | Was the researcher male or female? | Methods, 1.2.6 Ensuring Rigor |
| Experience and training | 5 | What experience or training did the researcher have? | Methods, 1.2.4 Data Analysis Methods; 1.2.6 Ensuring Rigor |
| Relotionship with participants | | | |
| Relationship established | 6 | Was a relationship established prior to study commencement? | Methods, 1.1 Study Subjects; 1.2.6 Ensuring Rigor |
| Participant knowledge of the interviewer | 7 | What did the participants knowabout the researcher?eg personal goals,reasons for doing the research | Methods, 1.2.2 Data Collection Methods and Quality Control; 1.2.6 Ensuring Rigor |
| Interviewer characteristics | 8 | What characteristics were reported about the inter viewer/failitator? e.g.Bias,assumptions,reasons and interests in the research topic | Methods, 1.2.6 Ensuring Rigor |
| **Domain** **2:Study** **design** | | | |
| Theoretical framework | | | |
| Methodological orientation and Theory | 9 | What methodological orientation was stated to underpin the study?e.g grounded theory,discourse analysis,ethnography,phenomenology,content analysis | Methods, 1.2.1 Research Methods and Philosophical Foundations; Abstract |
| Participant selection | | | |
| Sampling | 10 | How were participants selected?e.g purposive,convenience, consecutive,snowball | Methods, 1.1 Study Subjects |
| Method of approach | 11 | How were participants approached?e.g.face-to-face,telephone,mail, email | Methods, 1.1 Study Subjects; 1.2.2 Data Collection Methods and Quality Control |
| Sample size | 12 | How many participants were in the study? | Abstract; Methods, 1.1 Study Subjects; Table 1 |
| Non-participation | 13 | How many people refused to participate or dropped out?Reasons? | Methods, 1.1 Study Subjects |
| Setting | | | |
| Setting of data collection | 14 | Where was the data collected?eg.home,clinic,workplace | Methods, 1.2.2 Data Collection Methods and Quality Control |
| Presence of non- participants | 15 | Was anyone else present besides the participants and researchers? | Methods, 1.2.5 Ethical Considerations |
| Description of sample | 16 | What are the important characteritics of the sample?eg.demographic data,date | Methods, 1.1 Study Subjects; Table 1 |
| Dota collection | | | |
| Interview guide | 17 | Were questions,prompts,guides provided by the authors?Was it pilot tested? | Methods, 1.2.1 Developing the Interview Guide |
| Repeat interviews | 18 | Were repeat inter views carried out?Ifyes,how many? | Methods, 1.2.2 Data Collection Methods and Quality Control |
| Audio/visual recording | 19 | Did the research use audio or visual recording to collect the data? | Methods, 1.2.2 Data Collection Methods and Quality Control |
| Field notes | 20 | Were field notes made during and/or after the inter view or focus group? | Methods, 1.2.2 Data Collection Methods and Quality Control |
| Duration | 21 | What was the duration of the inter views or focus group? | Methods, 1.2.2 Data Collection Methods and Quality Control |
| Data saturation | 22 | Was data saturation discussed? | Methods, 1.1 Study Subjects; 1.2.4 Data Analysis Methods |
| Transcripts returned | 23 | Were transcripts returned to participants for comment and/or correction? | Methods, 1.2.2 Data Collection Methods and Quality Control |

| **Topic** | **Item** **No.** | **Guide** **Questions/Description** | **Reported** **on** **Page** **No.** |
| --- | --- | --- | --- |
| **Domain** **3:analysis** **and** **findings** | | | |
| Data analysis | | | |
| Number of data coders | 24 | How many data coders coded the data? | Methods, 1.2.4 Data Analysis Methods |
| Description of the coding tree | 25 | Did authors provide a description of the coding tree? | Methods, 1.2.4 Data Analysis Methods; Supplementary File2 |
| Derivation of themes | 26 | Were themes identified in advance or derived from the data? | Methods, 1.2.4 Data Analysis Methods |
| Software | 27 | Wha software,if applicable,was used to manage the data? | Methods, 1.2.4 Data Analysis Methods |
| Participant checking | 28 | Did participants provide feedback on the findings? | Methods, 1.2.4 Data Analysis Methods; 1.2.6 Ensuring Rigor |
| Reporting | | | |
| Quotations presented | 29 | Were participant quotations presented to illustrate the themes/findings? Was each quotation identified?e.g.participant number | Results, 2. Results |
| Data and findings consistent | 30 | Was there consistency between the data presented and the findings? | Results, 2. Results |
| Clarity of major themes | 31 | Were major themes clearly presented in the findings? | Results, 2.1; 2.2; 2.3 |
| Clarity of minor themes | 32 | Is there a description of diverse cases or discussion of minor themes? | Results, 2.1.1–2.3.2 |

Developed from:Tong A,Sainsbury P,CraigJ.Consolidated criteria for reporting qualitative research (COREQ):a 32-item checklist for interviews and focus groups.Internotional Journalfor Quality in Heolth Care.2007.Volume 19Number 6:p.349-357

**Once you have completed this checklist,please save a copy and upload it as part of your submission.DO NOT include thi**s **checklist as part of the main manuscript document.It must be uploaded as a separate file.**
